# Downregulation of the Netrin-1 Receptor UNC5b Underlies Increased Placental Angiogenesis in Human Gestational Diabetes Mellitus

**DOI:** 10.3390/ijms20061408

**Published:** 2019-03-20

**Authors:** Catalina P. Prieto, Bárbara S. Casas, Paulina Falcón, Andrea Villanueva, Pablo Lois, José Lattus, Verónica Palma

**Affiliations:** 1 Laboratory of Stem Cells and Developmental Biology, Faculty of Sciences, Universidad de Chile, Santiago de Chile 7800024, Chile; cpprieto@uchile.cl (C.P.P.); barbara.s.casas@gmail.com (B.S.C.); paulina.falcon.u@gmail.com (P.F.); andreavillanuevaarros@gmail.com (A.V.); pablois@uchile.com (P.L.); 2 Campus Oriente, Department of Obstetrics and Gynecology, Faculty of Medicine, Universidad de Chile, Santiago de Chile 7800024, Chile; doctorjoselattus@gmail.com

**Keywords:** GDM, UNC5b, angiogenesis, Netrin-1, HUVEC, WJ-MSC

## Abstract

Gestational diabetes mellitus (GDM) is a common metabolic disorder, defined by high blood glucose levels during pregnancy, which affects foetal and post-natal development. However, the cellular and molecular mechanisms of this detrimental condition are still poorly understood. A dysregulation in circulating angiogenic trophic factors, due to a dysfunction of the feto-placental unit, has been proposed to underlie GDM. But even the detailed study of canonical pro-angiogenic factors like vascular endothelial growth factor (VEGF) or basic Fibroblast Growth Factor (bFGF) has not been able to fully explain this detrimental condition during pregnancy. Netrins are non-canonical angiogenic ligands produced by the stroma have shown to be important in placental angiogenesis. In order to address the potential role of Netrin signalling in GDM, we tested the effect of Netrin-1, the most investigated member of the family, produced by Wharton’s Jelly Mesenchymal Stem Cells (WJ-MSC), on Human Umbilical Vein Endothelial Cells (HUVEC) angiogenesis. WJ-MSC and HUVEC primary cell cultures from either healthy or GDM pregnancies were exposed to physiological (5 mM) or high (25 mM) d-glucose. Our results reveal that Netrin-1 is secreted by WJ-MSC from healthy and GDM and both expression and secretion of the ligand do not change with distinct experimental glucose conditions. Noteworthy, the expression of its anti-angiogenic receptor UNC5b is reduced in GDM HUVEC compared with its expression in healthy HUVEC, accounting for an increased Netrin-1 signalling in these cells. Consistently, in healthy HUVEC, UNC5b overexpression induces cell retraction of the sprouting phenotype.

## 1. Introduction

Gestational Diabetes Mellitus (GDM) is a metabolic disorder defined by high blood glucose levels. GDM develops during pregnancy and usually disappears after giving birth [1]. This condition can cause alterations in the mother including gestational hypertension, metabolic syndrome and increased risk for type 2 diabetes. On the other hand, the effects in the developing foetus include macrosomia, hypoglycaemia, respiratory distress syndrome, hyperbilirubinemia, among others. However, with accurate clinical management of GDM through periodic screening for hyperglycaemia and intervening patient blood glucose levels (e.g., by healthy diet, exercise and healthy weight) some of these complications can be reduced [2].

It is well established that an imbalance in angiogenesis is implicated in different pregnancy disorders. In GDM, as a consequence of a dysfunction of the feto-placental unit, an increased angiogenesis, due to an increase in the mean number of redundant connections per villus compared to those in control subjects, has been reported [3,4,5]. However, the participation of canonical angiogenic molecules, such as vascular endothelial growth factor (VEGF) and basic Fibroblast Growth Factor (bFGF) and their receptors in this placental adaptation is unclear [1,5,6]. Thus, to date it is still unknown which signalling molecules promote abnormal activation of the endothelial cell, contributing to this pathological condition.

Netrin-1 is a secreted protein, which was first described as a neuronal guidance factor [7]. More recently, Netrin-1 has been shown to act as multifunctional protein involved in many cellular events, including cell polarity, adhesion, migration and angiogenesis. Interestingly, Netrin-1 has been involved in the regulation of placentation and, consequently, could play an important role in foetal growth [8]. Due to the fact that GDM has significant consequences in metabolic programming for women and their offspring, for both short and longer-term periods and considering that Netrin-1 governs growth and angiogenesis of various non-neuronal tissues, we reasoned that Netrin-1 signalling might be relevant in this context.

Netrin-1 has been associated to promotion or repression of angiogenesis in diverse vascular beds, depending on its association to at least 8 different receptors: Deleted in Colorectal Cancer (DCC), Neogenin-1, the Uncoordinated5 (UNC5) family comprising UNC5a-d and α6β4 and α3β4 integrins [9,10]. Classically, Netrin-1 dependent chemoattraction is mediated by the DCC receptor, while the UNC5 receptors mediate axonal repulsion [11,12]. Recently, we showed that the Netrin-1 signalling pathway modulates angiogenesis in the human umbilical cord and that members of the UNC5 family are expressed in primary cultures of HUVEC [13]. However, in both foetal endothelium and stromal cells, we did not detect any expression of DCC [13]. Both Netrin-1 and UNC5b are expressed by placental and endothelial cells during development and are important for proper placental vascular growth [8]. The latter suggests that a potential activation of UNC5 receptors by Netrin-1 could inhibit sprouting angiogenesis in the foetal-placental vasculature.

The connective tissue of the umbilical cord, called Wharton’s Jelly (WJ), surrounds the foetal vessels and is thought to prevent compression, torsion and bending [14]. This tissue is rich in mesenchymal stem cells (WJ-MSC), which secrete a broad set of molecules that participate in migration, inflammatory response, angiogenesis, tissue repair and coagulation [15]. Among the angiogenic factors, WJ-MSC secrete canonical pro- and anti-angiogenic molecules [15], as well as non-canonical pro-angiogenic factors like Netrin-1. Here, we intended to characterize the role of the Netrin-1 signalling pathway in GDM by evaluating Netrin-1 secretion in WJ-MSC and its receptors profiling in HUVEC. In order to mimic the hyperglycaemic environment observed in GDM, we performed the experiments exposing both healthy and GDM WJ-MSC and HUVEC cultures to physiological (5 mM ~ 90 mg/dL blood glucose) or high (25 mM ~ 450 mg/dL blood glucose) d-glucose concentration for up to 48 h [16].

The present study highlights the participation of a new signalling pathway, Netrin-1/UNC5b, that could be involved in the pathogenesis of GDM, with possible therapeutic relevance.

## 2. Results

### 2.1. Healthy and GDM WJ-MSC Express and Secrete Similar Amounts of Netrin-1

**Figure 1:**
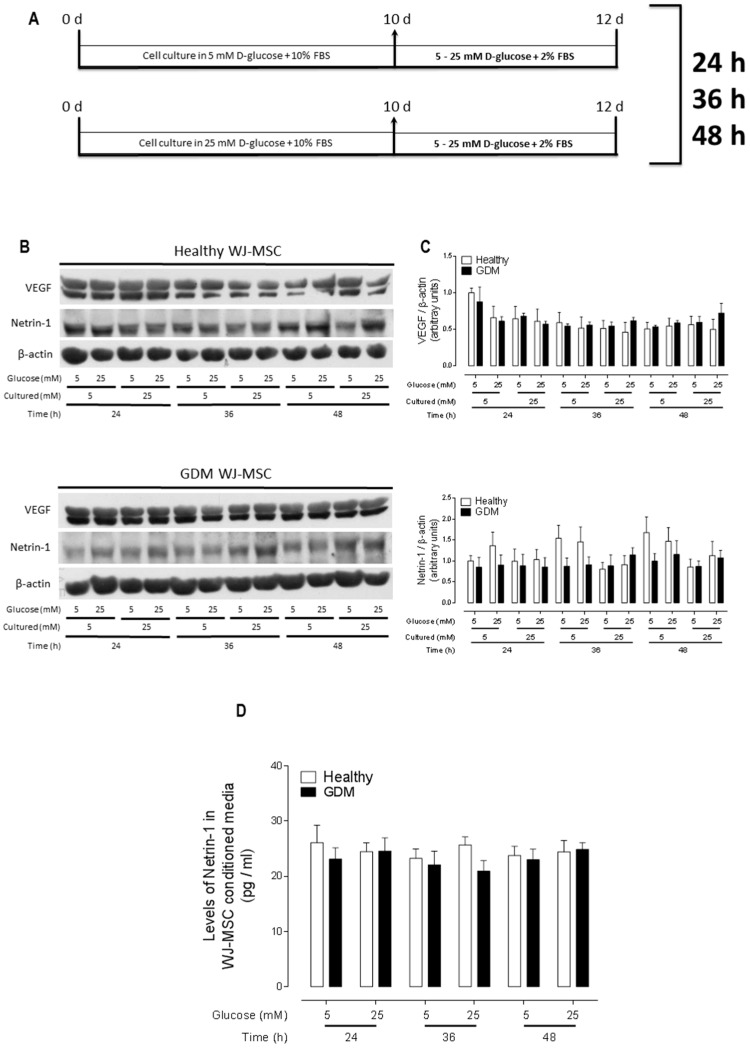
Netrin-1 expression and secretion do not change in healthy versus GDM WJ-MSC. (**A**) Scheme of the experimental procedures. WJ-MSC primary cultures were maintained for 10 days at the indicated glucose concentration in 10% FBS and then incubated for up to 2 more days in either 5 mM or 25 mM plus 2% FBS. Samples were collected at indicated times for analysis. The vertical arrow indicates initiation of differential d-glucose treatment. (**B**) Whole cell lysates were evaluated by Western blot for Netrin-1 expression in WJ-MSC from healthy and GDM cultures. VEGF was used as a positive control and β-actin was used as internal loading control. Exposure to different d-glucose concentration as indicated. (**C**) Data represent the mean of independent biological replicates ± SEM (healthy *n* = 12, GDM *n* = 6). (**D**) Quantitation of Netrin-1 by Elisa analysis. CM from healthy and GDM WJ-MSC was obtained to evaluate Netrin-1, CM was obtained with different d-glucose treatment at indicated time points (*n* = 4).

VEGF and other canonical pro-angiogenic proteins (such as bFGF) have not been involved in the increased angiogenesis observed in GDM pathology and thus do not represent reliable candidates for early detection nor risk factors to develop GDM. Indeed, concordantly with previous data, we found that VEGF protein expression was similar among healthy and GDM WJ-MSC, with comparable protein levels even when cells were exposed to 25 mM d-glucose media (thus mimicking the diabetic niche) for 24–48 h. Regarding to non-canonical pro-angiogenic proteins such as Netrin-1, we did not find any difference in its expression levels in healthy versus GDM WJ-MSC, neither when exposed to 5 mM or 25 mM d-glucose media (Figure 1B,C). Of note, we also measured the protein expression levels of Netrin-1 receptors Neogenin-1 and UNC5b, being similar between healthy and GDM WJ-MSC, both in 5 mM and 25 mM d-glucose culture conditions, over the time period studied (Supplementary Figure S1A,B). In addition, we evaluated if the secretion of Netrin-1 was altered in GDM WJ-MSC or modulated by glucose levels, for which we measured Netrin-1 levels by ELISA in healthy and GDM WJ-MSC CM after exposition to 5 mM or 25 mM d-glucose media. Netrin-1 levels were constant with concentrations ranging ~25–30 pg/mL, which is similar to the recently reported levels of Netrin-1 in the umbilical cord blood [17]. Thus, Netrin-1 secretion was not modulated by glucose exposure (Figure 1D). It is noteworthy that we have shown previously that another Netrin ligand, Netrin-4, is not expressed by WJ-MSC [13].

### 2.2. WJ-MSC’s Netrin-1 Has an Angiogenic Effect in GDM-HUVEC

**Figure 2:**
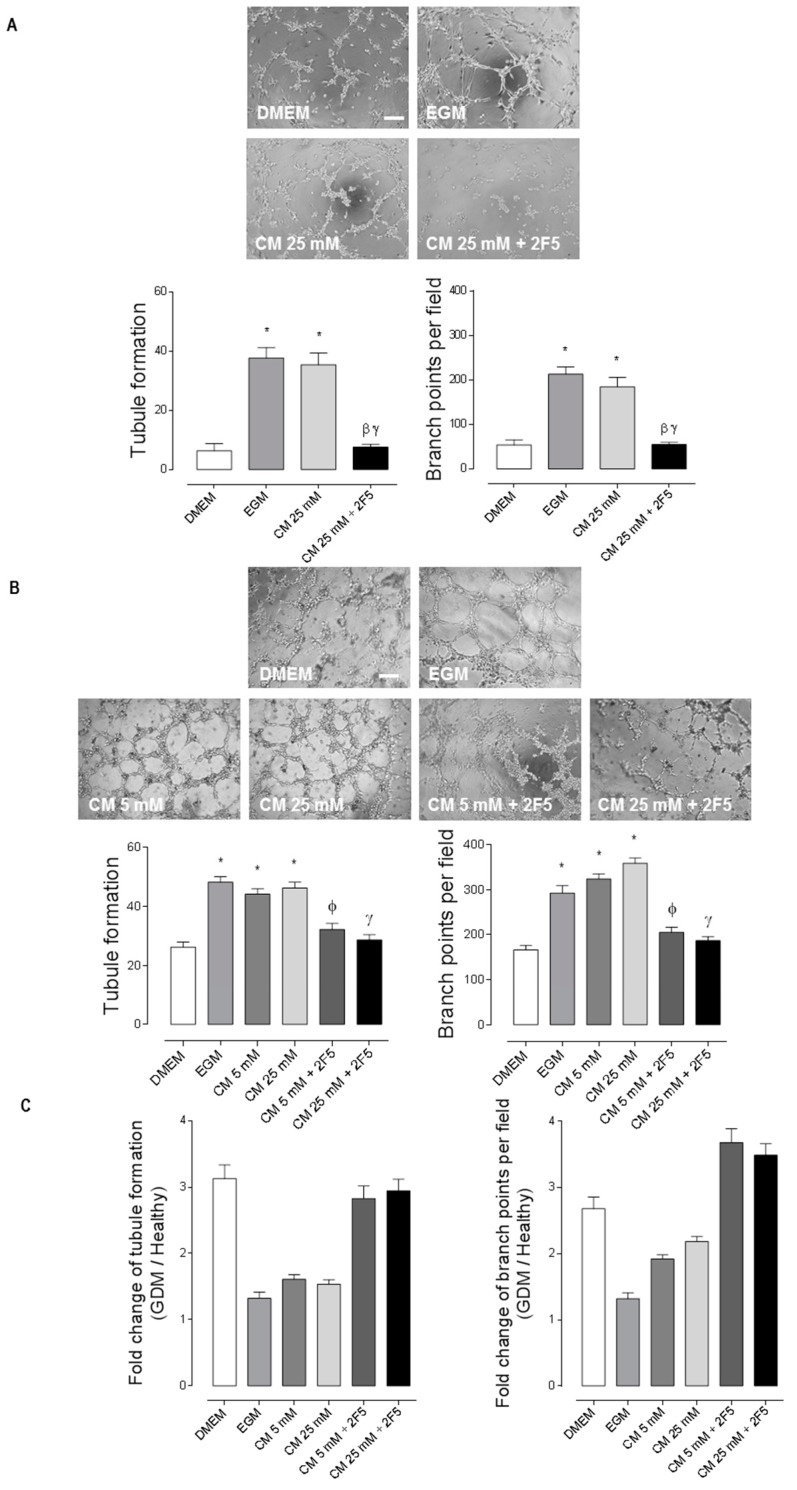
Netrin-1 produced by healthy WJ-MSC promotes angiogenesis in GDM HUVEC independently of d-glucose condition. (**A**–**C**) Representative images of Matrigel tubule formation and branch points per field of either normal HUVEC (**A**) or GDM HUVEC (**B**) exposed 4 h to DMEM, EGM or CM (48 h) from WJ-MSC grown in d-glucose as indicated and in absence or presence of 2F5 (2 µg/mL). Scale bar = 15 µm. ***A.*** Quantified data correspond to the mean ± S.E.M. (CM WJ-MSC and healthy HUVEC, *n* = 3, * *p* < 0.05 vs. DMEM, β*p* < 0.05 vs. EGM, γ*p* < 0.05 vs. CM 25 mM). (**B**) Quantified data correspond to the mean ± S.E.M. (CM WJ-MSC and GDM HUVEC, *n* = 3, * *p* < 0.05 vs. DMEM, Φ*p* < 0.05 vs. CM 5 mM, γ*p* < 0.05 vs. CM 25 mM). (**C**) Fold change of tubule formation assay in healthy versus GDM HUVEC. Quantified data correspond to the mean ± S.E.M.

Since secretion of Netrin-1 was not altered in GDM WJ-MSC CM compared to healthy WJ-MSC CM, we evaluated if WJ-MSC CM has a functional difference in the angiogenic response of both healthy and GDM HUVEC. Corroborating our previous data [13], CM secreted in 5 mM d-glucose medium for 48 h by WJ-MSC, induced tube and ramification branch points formation in healthy HUVEC. We did not find any difference in the angiogenic response in healthy HUVEC exposed to CM from WJ-MSC cultured in 5 mM [13] versus 25 mM glucose conditions. Regardless of the d-glucose concentration of the CM, we found that this pro-angiogenic response was largely dependent on Netrin-1 secreted by WJ-MSC, since we observed a ~70% reduction in the response when using the recombinant anti-Netrin-1 antibody (2F5, 2 µg/mL) (Figure 2A). The same approach was used to study the pro-angiogenic effect of Netrin-1 in GDM HUVEC. We observed an increased basal angiogenesis in GDM HUVEC compared to healthy HUVEC (Compare Figure 2A and Figure 2B), forming ~3 times more both tubes and branching points in control conditions (DMEM) and ~1.3 in angiogenic conditions (EGM). We corroborated a Netrin-1 dependency in the angiogenic response of GDM HUVEC, although notably, the effect was subtle compared to healthy HUVEC, assessing a ~30% and ~40% reduction in angiogenic response when adding the recombinant antibody anti-Netrin-1 2F5 in 5 mM and 25 mM d-glucose culture conditions, respectively. The latter suggests that other trophic factors or receptors might be implicated in this increased GDM angiogenesis. We tabulated the statistical differences of the angiogenic response in vitro between healthy and GDM HUVEC (Supplementary Figure S2). In addition, we graphed the fold change of each condition assayed, evidencing that there is an increased angiogenesis in GDM HUVEC compared to healthy HUVEC in the basal (DMEM) condition and when blocking Netrin-1 with 2F5 in both 5 mM and 25 mM d-glucose WJ-MSC’s CM (Figure 2C).

### 2.3. Downregulation of the Anti-Angiogenic UNC5b Receptor Could Explain the Increased Angiogenesis Observed in GDM HUVEC

**Figure 3:**
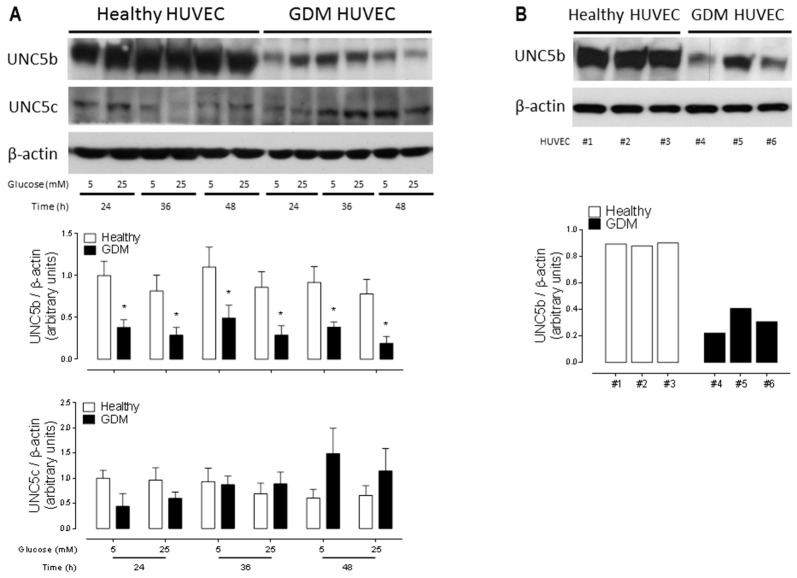
The classical anti-angiogenic Netrin-1 receptor UNC5b is decreased in GDM HUVEC. (**A**) Upper panel shows a representative Western blot for UNC5b and UNC5c protein expression levels in healthy and GDM HUVEC cell lysates. β-actin was used as internal loading control. d-glucose concentration and time points as indicated. Graphs below show quantified results corresponding to the mean ± S.E.M. (healthy *n* = 3, GDM *n* = 3). * *p* < 0.05 vs. healthy. (**B**) Different HUVEC from fresh umbilical cords randomly selected are indicated as #1-6. Western Blot revealing UNC5b protein expression levels in primary cell cultures (0–1 passages) of |healthy and GDM HUVEC. β-actin was used as internal loading control. Graph below shows receptor expression per replicate, labelled #1, #2, #3 for healthy and #4, #5, #6 for GDM HUVEC.

Although HUVEC secrete canonical and non-canonical angiogenic trophic factors (Supplementary Figure S3) an autocrine secretion of Netrins does not induce a significant angiogenic response in vitro [13]. Considering that the influence of secreted Netrin-1 by WJ-MSC in either 5 mM or 25 mM d-glucose is positive, resulting in a pro-angiogenic stimulus, we next focused on the expression of classical Netrin-1 receptors in HUVEC. As previously described, DCC is not detected while Neogenin-1 is expressed at low levels in these cells [13]. Interestingly, when evaluating the expression of the anti-angiogenic UNC5b receptor we found that its expression was significant reduced in GDM HUVEC regardless of culturing in 5 mM or 25 mM d-glucose (Figure 3A). We corroborated this phenomenon even in GDM HUVEC of early passages (0-1) (Figure 3B). Noteworthy, another member of the family, the UNC5c receptor, does not change its expression levels in healthy versus GDM HUVEC and when cultured under different d-glucose treatments (Figure 3A).

### 2.4. Overexpression of the Anti-Angiogenic UNC5b Receptor Reduces Angiogenesis In Vitro

**Figure 4:**
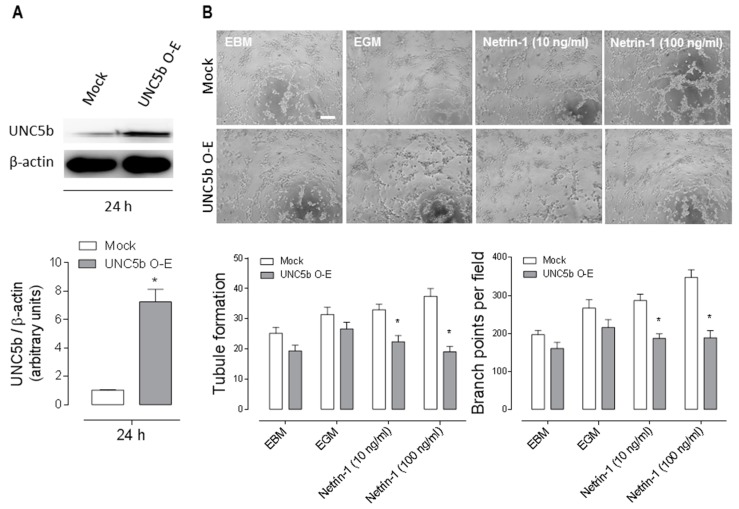
Overexpression of the anti-angiogenic receptor UNC5b prevents the pro-angiogenic response of Netrin-1 in HUVEC. Healthy HUVEC were transfected with UNC5b O-E or Mock-expressing constructs (1 μg/well) (**A**) To confirm UNC5b overexpression, cell lysates were analysed by Western Blot. β-actin levels were determined as loading control. Data correspond to the mean ± S.E.M (healthy *n* = 3). * *p* < 0.05 vs. internal control. (**B**) The overexpression of UNC5b was evaluated through a 4 h of Matrigel tubule formation assay in presence of Netrin-1. Representative images of treatments as indicated. Data correspond to the mean ± S.E.M (healthy *n* = 3). Scale bar = 15 µm. * *p* < 0.05 vs. internal control.

Since the anti-angiogenic receptor UNC5b was decreased in GDM HUVEC, we asked whether high levels of UNC5b may be inhibitory for angiogenesis. To test that, we transfected healthy HUVEC with an UNC5b construct. As reported [18], HUVEC are difficult to transfect; with our protocol, we detected a significant reduction of cell survival (~85% cell death) after construct transfection (Supplementary Figure S4). Immunoblotting confirmed a high expression of UNC5b in the transfected cells (Figure 4A). After 24 h of transfection, we performed a tube formation assay exposing the cells to different experimental conditions for 4 h. UNC5b overexpression had no significant effect on angiogenesis in basal conditions (EBM) or in the positive control (EGM) media. Likewise, the addition of human recombinant Netrin-1 at physiological concentrations (10–100 ng/mL) had a pro-angiogenic effect in control HUVEC with similar levels to EGM. Strikingly, when overexpressing UNC5b, human recombinant Netrin-1 at 10–100 ng/mL promoted an anti-angiogenic effect. The latter most probably due to an activation of the anti-angiogenic UNC5b receptor by Netrin-1 (Figure 4B) which would be in line with the role ascribed for this receptor in angiogenesis [19]. Overall our results suggest that the increased angiogenesis observed in GDM HUVEC could be at least partially explained by a lower expression of the anti-angiogenic receptor UNC5b, contributing to the etiopathogenesis of GDM.

## 3. Discussion

GDM is a metabolic disorder in a heterogeneous group of women that develop pregnancy-associated risk. GDM is usually detected in the late 2nd gestational trimester or third trimester. Its diagnosis and treatment are under continuous debate. However, despite this controversy, there is substantial evidence that there is not only one risk factor to address this pathology [20].

Feto-placental angiogenesis predominates during the third trimester of pregnancy [3]. This mechanism occurs as new capillaries are formed by sprouting of already existing villous vessels, allowing the development of mature intermediate and terminal villi [21]. Thus, GDM may have an impact on placental processes occurring in later stages of pregnancy, such as microvascular remodelling and will not affect developmental events that occur in early pregnancy, such as vasculogenesis. It is important to mention that, although GDM is responsible for fewer birth defects than pre-gestational diabetes, is well known that the mean number of redundant connections per villus is increased in GDM. The latter can lead to foetal complications, such as macrosomia and hypoglycaemia, as well as maternal complications, including hypertension, preeclampsia and an increased risk of Caesarean delivery in GDM [3]. At present, the cellular and molecular mechanisms that trigger and predict the evolution of this disease remain mostly unknown.

Proper feto-placental development depends on different signalling factors, however the endothelial signalling machinery that mediates the establishment of the placental vasculature is largely unclear [22]. Netrin-1 has been described as a key molecule in many different contexts, ranging from cancer progression [23], branching and membrane extension on oligodendrocytes [24], axon outgrowth in neurons [25] to osteoclast differentiation in bone [26] and angiogenesis [10]. Remarkably, as per our knowledge, this is the first study showing a characterization of the bioactivity of a non-canonic pro-angiogenic factor like Netrin-1 and its receptors during pregnancy and their potential involvement in the GDM pathology.

We have shown previously that both Netrin-1 and its receptors are differentially expressed in healthy HUVEC and WJ-MSC [13]. Similar results were found in HUVEC or WJ-MSC from GDM exposed to different d-glucose concentrations. Netrin-1 is secreted by WJ-MSC from healthy and GDM and both expression and secretion of the ligand do not change with distinct experimental glucose conditions. This result is in disagreement with previous studies performed on other types of diabetes, where researchers have reported that the levels of Netrin-1 are reduced in human type 2 diabetes mellitus [27] or in streptozotocin-induced type 1 diabetes in mice [28].

Despite of not finding differences in terms of Netrin-1 secretion, we observed significant differences in the GDM HUVEC angiogenic response versus healthy HUVEC when exposed to healthy WJ-MSC CM. This pro-angiogenic effect of the ligand in GDM HUVEC could be found in all experimental conditions tested but was particularly notorious in the relative basal angiogenic response.

Netrin-1′s effect in healthy HUVEC was in agreement with another report showing the same effect [29]. Noteworthy, using a recombinant anti-Netrin-1 antibody (2F5), we detected that Netrin-1 pro-angiogenic contribution was more significant in healthy (~70%) than in GDM (~40%) HUVEC. Importantly, this is the first time that antibody mediated blockage of Netrin-1 was used in the context of the GDM pathology, lowering GDM HUVEC’s angiogenic response.

Given that a differential pro-angiogenic contribution of Netrin-1 was not enough to explain the increase in basal angiogenesis observed in GDM compared to healthy HUVEC, we evaluated a possible differential response of the endothelial cells, in particular focusing on the different classical receptors for Netrin-1. Netrin-1 acts as a chemorepulsant and inhibits angiogenesis by activation of its receptor UNC5b. Interestingly, we recently reported that healthy HUVEC express the anti-angiogenic receptor UNC5b [13]. Hence, UNC5b emerged as a possible candidate to explain the differential angiogenic response that could contribute to initiation and/or maintenance of this pathology during pregnancy. In fact, when we evaluated the total UNC5b expression in endothelial cells we found a decreased expression of this receptor in GDM versus healthy HUVEC. Along this line, it has been previously shown that, at full-term pregnancy, UNC5b is observed in human decidual cells, which are located in the maternal side with less expression in extravillous cells located on the foetal side [8]. Recently, it has been demonstrated in rodents that the formation of the placental labyrinth, which is important in adequate gas and nutrient exchange at the interface between the foetus and the mother, requires endothelial Fibronectin Leucine-Rich Transmembrane protein (2FLRT2) acting as a repulsive ligand of UNC5b signalling [22]. However, is it is unknown if 2FLRT2 is expressed in human placenta. If expressed, it could act in collaboration with Netrin-1 for a fine-tuning of these processes. Besides, in accordance with our results, UNC5b has been described with high expression in healthy endothelial cells in the labyrinth [11]. Hence, our findings in GDM, showed a diminished expression of UNC5b in HUVEC, supporting a functional role of a Netrin-1/UNC5b signalling complex. Recent data have implicated another UNC5 receptor, UNC5H2, in insulin-resistance in obesity-driven diabetes [30]. Furthermore, it has been described that proteolytic fragments of Netrin-1, signalling trough UNC5b, can increase the vascular permeability in diabetic retinopathy [31], a phenomenon that we cannot rule out in our system.

To reinforce our findings regards UNC5b, we overexpressed the receptor on healthy HUVEC. We found that the addition of human recombinant Netrin-1 at physiological range promoted an anti-angiogenic effect due to the specific activation of UNC5b receptor, demonstrating that this receptor plays an important role in the angiogenesis processes. Noteworthy, it has been demonstrated that Netrin-1 promotes the survival of endothelial cells via UNC5b, since this receptor in its unbound state participates in the apoptosis process acting as a death dependence factor [32,33]. Altogether, considering that WJ-MSC contributes in Netrin-1 secretion and healthy versus GDM HUVEC have differential UNC5b expression, our data strongly suggest a relevant contribution of the Netrin-1/UNC5b signalling complex in the increased angiogenic response observed in GDM. Numerous studies have underlined the importance of Netrin signalling for blood vessel formation and remodelling, both under physiological and pathological conditions [10]. The effect of Netrin-1 in the vascular system is linked to differential Netrin receptor expression. To note, a new receptor for Netrin-1, CD146, which induces angiogenesis in endothelial cells [29], has recently been reported. We found that HUVEC express low levels of Netrin-1 when compared to Netrin-4. Due to this observation, we propose that the pro-angiogenic contribution comes from WJ-MSC’ Netrin-1, which secrete more Netrin-1 than Netrin-4 [13]. Netrin-1 signalling induces UNC5B mediated downstream activation of signalling pathways like PI3K, ERK and other unknown pathways. Activation of PI3K is known to stimulate the protein kinase AKT/NOS/NO signalling pathway leading to increased angiogenesis [34,35].

**Figure 5:**
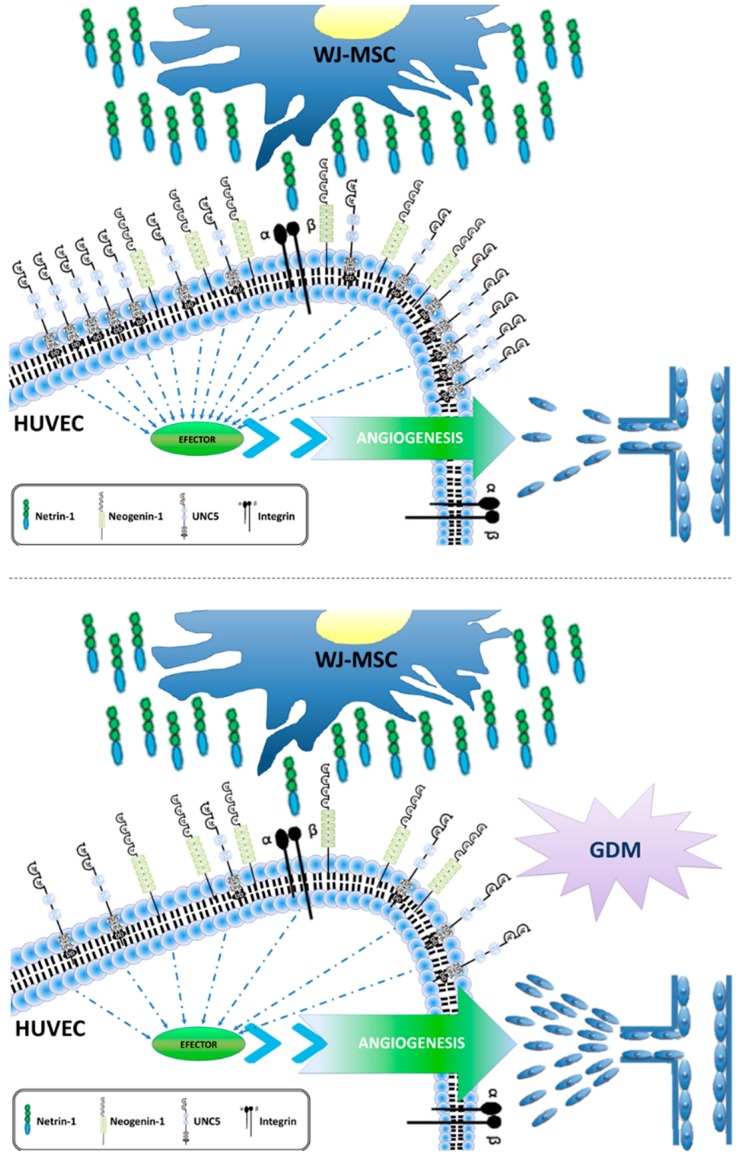
Working Model. Model integrating the pro-angiogenic effect of Netrin-1 secretion by WJ-MSC. Endothelial cells express both classical UNC5b and UNC5c, as well as non-classical receptors (Integrins) for Netrin-1. Considering that UNC5b is an anti-angiogenic receptor, it is feasible that loss of UNC5b in endothelial cells promotes alterations in placental angiogenesis, as observed in GDM.

This research is an extension of previous work in which we demonstrated that Netrin-1 is a critical paracrine regulator of angiogenesis and signals independent of the RhoA/ROCK signalling pathway [13]. Here we further extended the observation that Netrin-1 is highly expressed by WJ-MSC and binds to its anti-angiogenic receptor UNC5b located in adjacent endothelial cells, which has a lower expression in GDM, suggesting a relevant contribution to develop this pathology (Figure 5). Therefore, it is relevant to consider that the stromal/endothelial niche is essential to preserve functional placental angiogenesis for the maintenance of normal foetal development and that maternal diet/exercise as treatment of GDM is not sufficient to change the foetal metabolic derangement during pregnancy, because the foetal programming is maintained.

At the moment, we cannot rule out that in addition to a reduced anti-angiogenic UNC5b receptor presence in HUVEC other key angiogenic receptors participate in this altered endothelial phenotype. Recent data suggest that GDM is associated with reduced expression of VEGF receptor Flt-1 but increased KDR expression levels in GDM placentas, thus supporting a proangiogenic state in GDM [36]. In line with this, the same study reported a significant increase in cell migration in GDM HUVEC when compared to healthy HUVEC. Altogether, the final angiogenic imbalance in GDM could thus be the result of more than one signalling cascade, a matter that deserves further investigation.

A decrease of Netrin-1signaling in placenta of patients with pre-eclampsia has been postulated as one of the possible reasons for the reduction of placenta vascular density [37]. Moreover, Qian-Hua et al. demonstrated that Netrin-1 expression was significantly reduced in placenta from women bearing foetuses with growth restriction when compared to pregnant control women [38]. These data provide additional arguments to deepen studies regarding Netrin-1 signalling as a potential target for the development of new therapeutic strategies in placental-vasculature-related diseases.

## 4. Materials and Methods

### 4.1. Isolation and Cell Culture of WJ-MSC and HUVEC

The diagnosis of GDM was based on criteria from the World Health Organization (WHO) and performed by clinical staff from Dr. Luis Tisné Brousse Hospital, Santiago, Chile. Exclusions included (1) patients prescribed any oral hypoglycaemic agents or insulin, (2) impaired glucose tolerance or impaired fasting glucose, (3) a history of acute diabetic complications (4) severe uncontrolled hypertension, (5) any acute inflammation or infection (6) current or a history of significant comorbid diseases. The healthy group had normal glucose tolerance at the baseline examination and offered to participate voluntarily in this study. Healthy pregnancies were stablished as: non-smokers, normotensive, normal cholesterol levels, not having pre-eclampsia, pre-gestational nor GDM, no family history of premature vascular diseases and no regular consumption of medication [16]. Independent ethics committees from University of Chile, Dr. Luis Tisné Brousse Hospital and Servicio de Salud Metropolitano Oriente (SSMO) approved the study. Written informed consent from all patients was obtained prior to be enrolled and samples obtained within 2–24 h after caesarean delivery. The summary of demographic samples for healthy donors and GDM patients is shown in Table 1.

For WJ-MSC isolation we followed standard procedures [13,15]. Briefly, the umbilical cord from full-term pregnancies was dissected to discard blood vessels, cut into 2 mm^2^ pieces and digested with collagenase I (1 μg/μL, Gibco by Life Technologies, Carlsbad, CA, USA) in phosphate buffered saline (PBS, in mM: NaCl 136, KCl 2.7, Na2HPO4 7.8, KH2PO4 1.5, pH 7.4) with gentle agitation at 37 °C during 16 h in order to disaggregate the tissue. The cells obtained by subsequent centrifugation (2000 rpm, 10 min) were then washed and seeded in DMEM (Life Technologies) containing 10% FBS (Hyclone, Logan UT, USA) with antibiotics (100 U/mL Penicillin/Streptomycin, Thermo Scientific, Waltham, MA, USA) and maintained in this condition for 24 h at 37 °C, 5% CO_2_. Adherent cells were incubated at 37 °C, 5% CO_2_, changing the medium every 2–3 days. All primary cultures of WJ-MSC were used between passages 2–5. HUVEC were obtained from full-term umbilical cords as previously described [13,15]. Briefly, umbilical veins were rinsed with warm (37 °C) PBS. Endothelial cells were isolated by collagenase (0.2 mg/mL) digestion and cultured (37 °C, 5% CO_2_) in primary cell medium (PCM) composed by medium 199 (M199, Gibco by Life Technologies, Carlsbad, CA, USA) supplemented with 10% NBCS (Gibco by Life Technologies, Carlsbad, CA, USA), 10% FBS (Gibco by Life Technologies, Carlsbad, CA, USA), 3.2 mM l-glutamine (Sigma, St. Louis, MO, USA) and 100 U/mL penicillin-streptomycin (Thermo Scientific, Waltham, MA, USA). The medium was changed every 2 days until confluence was reached. All primary cultures of HUVEC were used between passages 2–5.

In order to mimic the hyperglycaemic microenvironment observed in GDM, both GDM and healthy cells were cultured at all times using an acute or chronic experimental treatment of d-glucose. For experimental procedures, WJ-MSC and HUVEC, were maintained in physiological (5 mM ~ 90 mg/dL blood glucose) or high (25 mM ~ 450 mg/dL blood glucose) d-glucose concentration for up to 48 h [28] (Figure 1A).

### 4.2. Tube Formation Assay

A tube formation assay was used to evaluate endothelial migration in healthy or GDM HUVEC. Matrigel (BD Matrigel; BD Biosciences Co., Bedford, MA, USA) was thawed overnight at 4 °C and administered by cold tips in 50 µL per well in cold 96-well cell culture plates. Matrigel made a thin gel layer after incubation at 37 °C for 1 h. 55,000 cells were seeded on each Matrigel-coated well and after 4 h, network formation was evaluated under a light microscope counting tube formation and ramifications points. Experimental conditions included conditioned media (CM) from healthy WJ-MSC cultured in either 5 mM or 25 mM d-glucose for 48 h, in absence/presence of 2F5, a neutralizing Netrin-1 antibody (2 µg/mL, Adipogen, Epalinges, Switzerland) or its isotypic control. In order to inhibit basal secretion of Netrin-1, HUVEC were cultured 1 h with 2F5 prior to each experiment. Results represent experiments carried out in duplicate at least three times.

### 4.3. Western Blot

Primary cell cultures from healthy and GDM HUVEC or WJ-MSC were harvested in 5 mM or 25 mM d-glucose for 24–48 h. Cell homogenates were suspended in SDS lysis buffer with added protease and phosphatase inhibitors at 4 °C. 60 µg of protein were separated by 8–12% SDS-PAGE, followed by Western blotting using overnight primary antibodies for anti-Netrin-1 (66 kDa) and -4 (70–75 kDa) (R&D Systems, Minneapolis, MI, USA), anti-VEGF (43–45 kDa) and anti-UNC5c (103 kDa) (Abcam, Cambridge, UK), anti-DCC (180–190 kDa) (BD Transduction Laboratories, CA, USA), anti-UNC5b (130 kDa) (Cell Signalling, Danvers, MA, USA) and anti-Neogenin-1 (175 kDa). Monoclonal mouse anti-β actin (42 kDa) (Sigma, St. Louis, MO, USA) antibody was used as loading control. Membranes were washed in Tris buffer saline (TBS) with 0.1% Tween and incubated (1 h, 22 °C) in TBS/0.1% Tween containing horseradish peroxidase-conjugated goat anti-rabbit, anti-sheep or anti-mouse secondary antibodies. Bands were visualized using enhanced chemiluminescence (ECL; Amersham Biosciences, Little Chalfont, UK). Western blot imaging was obtained with a UVITEC Imaging system (UVITEC Ldta., Cambridge, UK).

### 4.4. Netrin-1 Determination in Conditioned Media (CM)

In order to evaluate the secretion of Netrin-1 by WJ-MSC, CM were collected after 24–48 h in in serum starvation in either 5 mM or 25 mM of d-glucose culture media and immediately thawed in liquid nitrogen and stored −80°C. The CM was used to evaluate Netrin-1 levels through ELISA assay (USCN Life Science Inc., Huston, TX, USA).

### 4.5. Overexpression of UNC5b and Tube Formation Assay

250,000 HUVEC were plated into each 6-well plate. After 24 h, cells were transfected using the Turbofect^®^ protocol, with UNC5b or Mock overexpressing constructs (1 μg/well, kindly provided by Dr. Mehlen, Université de Lyon, Centre Léon Bérard, Lyon, France). Overexpression of UNC5b was evaluated by Western Blot. The tube formation assay in absence or presence of recombinant human Netrin-1 (rhNetrin-1, R&D Systems, 100 ng/mL, Minneapolis, MI, USA) versus Endothelial Basal Media (EBM), along with the internal positive control Endothelial Growth Medium (EGM), was performed as described above.

### 4.6. Statistical Analysis

Values are mean ± S.E.M., where n indicates number of independent cell cultures isolated from different umbilical cords (*n* = 3–7). Comparisons between two and more groups were performed by means of Student’s unpaired t-test and analysis of variance (ANOVA), respectively. If the ANOVA demonstrated a significant interaction between variables, post hoc analyses were performed by multiple-comparison Bonferroni correction test. All the determinations were carried out in triplicate. The software GraphPad Prism 5.0b (GraphPad Software Inc, San Diego, CA, USA) was used for data analysis. *p* < 0.05 was considered statistically significant.

## 5. Conclusions

Here we show that WJ-MSC’s Netrin-1 secretion does not account for the increased angiogenesis in GDM, although UNC5b decreased protein abundance in GDM HUVEC might explain this phenotype. Despite of not finding differences in terms of Netrin-1 production and secretion, we observed significant differences in the GDM HUVEC angiogenic response versus healthy HUVEC when exposed to healthy WJ-MSC’s conditioned media. Thus, the stromal/endothelial niche is essential to maintain functional placental angiogenesis.

## Figures and Tables

**Table 1:** Demographic characteristics of study population.

Variables	Healthy (*n* = 40)	GDM (*n* = 25)
Age (years)	30 ± 7	31 ± 7
Height (m)	1.54 ± 0.04	1.55 ± 0.05
Weight (kg)	65.6 ± 6	63.7 ± 3
Body mass index (kg/m^2^)	27.8 ± 0.1	26.4 ± 0.2
Fasting blood glucose	78 ± 6.7	78.3 ± 3.7
Postprandial blood Glucose (2 h)	99 ± 16.7	151 ± 9.6 *

All parameters were expressed as mean ± SD. * *p* < 0.05 vs. healthy control group.

## Supplementary Materials

Supplementary materials can be found at https://www.mdpi.com/1422-0067/20/6/1408/s1.

## References

[1] Desoye G., Hauguel-de Mouzon S. (2007). The Human Placenta in Gestational Diabetes Mellitus. Diabetes Care.

[2] Reece E.A. (2010). The fetal and maternal consequences of gestational diabetes mellitus. J. Matern Neonatal Med. Taylor Francis.

[3] Jirkovská M., Kubínová L., Janáček J., Moravcová M., Krejčí V., Karen P. (2002). Topological Properties and Spatial Organization of Villous Capillaries in Normal and Diabetic Placentas. J. Vasc. Res..

[4] Zheng M.J.E.-J. (2012). The Morphology of Villous Capillary Bed in Normal and Diabetic Placenta. Rijeka IntechOpen.

[5] Huynh J., Dawson D., Roberts D., Bentley-Lewis R. (2015). A systematic review of placental pathology in maternal diabetes mellitus. Placenta.

[6] Sobrevia L., Abarzúa F., Nien J.K., Salomón C., Westermeier F., Puebla C., Cifuentes F., Guzmán-Gutiérrez E., Leiva A., Casanello P. (2011). Review: Differential placental macrovascular and microvascular endothelial dysfunction in gestational diabetes. Placenta.

[7] Serafini T., Kennedy T.E., Gaiko M.J., Mirzayan C., Jessell T.M., Tessier-Lavigne M. (1994). The netrins define a family of axon outgrowth-promoting proteins homologous to C. elegans UNC-6. Cell.

[8] Dakouane-Giudicelli M., Duboucher C., Fortemps J., Missey-Kolb H., Brulé D., Giudicelli Y., de Mazancourt P. (2009). Characterization and Expression of Netrin-1 and Its Receptors UNC5B and DCC in Human Placenta. J. Histochem. Cytochem..

[9] Park K.W., Crouse D., Lee M., Karnik S.K., Sorensen L.K., Murphy K.J., Kuo C.J., Li D.Y. (2004). The axonal attractant Netrin-1 is an angiogenic factor. Proc. Natl. Acad. Sci. USA.

[10] Larrieu-Lahargue F., Thomas K.R., Li D.Y. (2012). Netrin ligands and receptors: Lessons from neurons to the endothelium. Trends Cardiovasc. Med..

[11] Navankasattusas S., Whitehead K.J., Suli A., Sorensen L.K., Lim A.H., Zhao J., Park K.W., Wythe J.D., Thomas K.R., Chien C.-B. (2008). The netrin receptor UNC5B promotes angiogenesis in specific vascular beds. Development.

[12] Finci L.I., Krüger N., Sun X., Zhang J., Chegkazi M., Wu Y., Schenk G., Mertens H.D.T., Svergun D.I., Zhang Y. (2014). The crystal structure of netrin-1 in complex with DCC reveals the bifunctionality of netrin-1 as a guidance cue. Neuron.

[13] Prieto C.P., Ortiz M.C., Villanueva A., Villarroel C., Edwards S.S., Elliott M., Lattus J., Aedo S., Meza D., Lois P. (2017). Netrin-1 acts as a non-canonical angiogenic factor produced by human Wharton’s jelly mesenchymal stem cells (WJ-MSC). Stem Cell Res. Ther..

[14] Nanaev A.K., Kohnen G., Milovanov A.P., Domogatsky S.P., Kaufmann P. (1997). Stromal differentiation and architecture of the human umbilical cord. Placenta.

[15] Edwards S.S., Zavala G., Prieto C.P., Elliott M., Martínez S., Egaña J.T., Bono M.R., Palma V. (2014). Functional analysis reveals angiogenic potential of human mesenchymal stem cells from Wharton’s jelly in dermal regeneration. Angiogenesis.

[16] Westermeier F., Salomón C., Farías M., Arroyo P., Fuenzalida B., Sáez T., Salsoso R., Sanhueza C., Guzmán-Gutiérrez E., Pardo F. (2014). Insulin requires normal expression and signaling of insulin receptor A to reverse gestational diabetes-reduced adenosine transport in human umbilical vein endothelium. FASEB J. Fed. Am. Soc. Exp. Biol..

[17] Boutsikou T., Giotaki M., Gourgiotis D., Boutsikou M., Briana D.D., Marmarinos A., Baka S., Hassiakos D., Malamitsi-Puchner A. (2014). Cord blood netrin-1 and -4 concentrations in term pregnancies with normal, restricted and increased fetal growth. J. Matern Neonatal Med. Taylor Francis.

[18] Hunt M.A., Currie M.J., Robinson B.A., Dachs G.U. (2010). Optimizing transfection of primary human umbilical vein endothelial cells using commercially available chemical transfection reagents. J. Biomol. Tech..

[19] Larrivée B., Freitas C., Trombe M., Lv X., Delafarge B., Yuan L., Bouvrée K., Bréant C., Del Toro R., Bréchot N. (2007). Activation of the UNC5B receptor by Netrin-1 inhibits sprouting angiogenesis. Genes Dev..

[20] Mission J.F., Catov J., Deihl T.E., Feghali M., Scifres C. (2017). Early Pregnancy Diabetes Screening and Diagnosis: Prevalence, Rates of Abnormal Test Results, and Associated Factors. Obstet. Gynecol..

[21] Castellucci M., Kosanke G., Verdenelli F., Huppertz B., Kaufmann P. (2000). Villous sprouting: Fundamental mechanisms of human placental development. Hum. Reprod. Update.

[22] Tai-Nagara I., Yoshikawa Y., Numata N., Ando T., Okabe K., Sugiura Y., Ieda M., Takakura N., Nakagawa O., Zhou B. (2017). Placental labyrinth formation in mice requires endothelial FLRT2/UNC5B signaling. Development.

[23] Shimizu A., Nakayama H., Wang P., König C., Akino T., Sandlund J., Coma S., Italiano J.E., Mammoto A., Bielenberg D.R. (2013). Netrin-1 promotes glioblastoma cell invasiveness and angiogenesis by multiple pathways including activation of RhoA, cathepsin B, and cAMP-response element-binding protein. J. Biol. Chem..

[24] Rajasekharan S., Baker K.A., Horn K.E., Jarjour A.A., Antel J.P., Kennedy T.E. (2009). Netrin 1 and Dcc regulate oligodendrocyte process branching and membrane extension via Fyn and RhoA. Development.

[25] Antoine-Bertrand J., Ghogha A., Luangrath V., Bedford F.K., Lamarche-Vane N. (2011). The activation of ezrin-radixin-moesin proteins is regulated by netrin-1 through Src kinase and RhoA/Rho kinase activities and mediates netrin-1-induced axon outgrowth. Mol. Biol. Cell.

[26] Mediero A., Ramkhelawon B., Perez-Aso M., Moore K.J., Cronstein B.N. (2015). Netrin-1 is a critical autocrine/paracrine factor for osteoclast differentiation. J. Bone Miner. Res..

[27] Liu C., Ke X., Wang Y., Feng X., Li Q., Zhang Y., Zhu J., Li Q. (2016). The level of netrin-1 is decreased in newly diagnosed type 2 diabetes mellitus patients. BMC Endocr. Disord..

[28] Toque H.A., Fernandez-Flores A., Mohamed R., Caldwell R.B., Ramesh G., Caldwell R.W. (2017). Netrin-1 is a novel regulator of vascular endothelial function in diabetes. PLoS ONE.

[29] Tu T., Zhang C., Yan H., Luo Y., Kong R., Wen P., Ye Z., Chen J., Feng J., Liu F. (2015). CD146 acts as a novel receptor for netrin-1 in promoting angiogenesis and vascular development. Cell Res..

[30] Ramkhelawon B., Hennessy E.J., Ménager M., Ray T.D., Sheedy F.J., Hutchison S., Wanschel A., Oldebeken S., Geoffrion M., Spiro W. (2014). Netrin-1 promotes adipose tissue macrophage retention and insulin resistance in obesity. Nat. Med..

[31] Miloudi K., Binet F., Wilson A., Cerani A., Oubaha M., Menard C., Henriques S., Mawambo G., Dejda A., Nguyen P.T. (2016). Truncated netrin-1 contributes to pathological vascular permeability in diabetic retinopathy. J. Clin. Investig..

[32] Castets M., Coissieux M.-M., Delloye-Bourgeois C., Bernard L., Delcros J.-G., Bernet A., Laudet V., Mehlen P. (2009). Inhibition of Endothelial Cell Apoptosis by Netrin-1 during Angiogenesis. Dev. Cell..

[33] Kefeli U., Ucuncu Kefeli A., Cabuk D., Isik U., Sonkaya A., Acikgoz O., Ozden E., Uygun K. (2017). Netrin-1 in cancer: Potential biomarker and therapeutic target?. Tumor Biol..

[34] Lee J.H., Parveen A., Do M.H., Lim Y., Shim S.H., Kim S.Y. (2018). Lespedeza cuneata protects the endothelial dysfunction via eNOS phosphorylation of PI3K/Akt signaling pathway in HUVECs. Phytomedicine.

[35] Karar J., Maity A. (2011). PI3K/AKT/mTOR Pathway in Angiogenesis. Front. Mol. Neurosci..

[36] Troncoso F., Acurio J., Herlitz K., Aguayo C., Bertoglia P., Guzman-Gutierrez E., Loyola M., Gonzalez M., Rezgaoui M., Desoye G. (2017). Gestational diabetes mellitus is associated with increased pro-migratory activation of vascular endothelial growth factor receptor 2 and reduced expression of vascular endothelial growth factor receptor 1. PLoS ONE.

[37] Yang Y., Zou L., Xu K.S. (2006). Expression of netrin-1 in placenta from patients with pre-eclampsia and the relation to placental angiogenesis. Zhonghua Fu Chan Ke Za Zhi.

[38] Qian-hua W., Shao-ping Z., Jian-wen Z., Yun Y., Li Z. (2011). Reduced expression of netrin-1 is associated with fetal growth restriction. Mol. Cell Biochem..

