# Supplementary material for: Downregulation of the Netrin-1 Receptor UNC5b Underlies Increased Placental Angiogenesis in Human Gestational Diabetes Mellitus
Source: Int J Mol Sci. 2019 Mar 20;20(6):1408. doi: 10.3390/ijms20061408 (PMC6470495; doi:10.3390/ijms20061408)
Supplement: Supplementary file 1 [file ijms-20-01408-s001.pdf]

SUPPLEMENTARY FIGURE LEGENDS

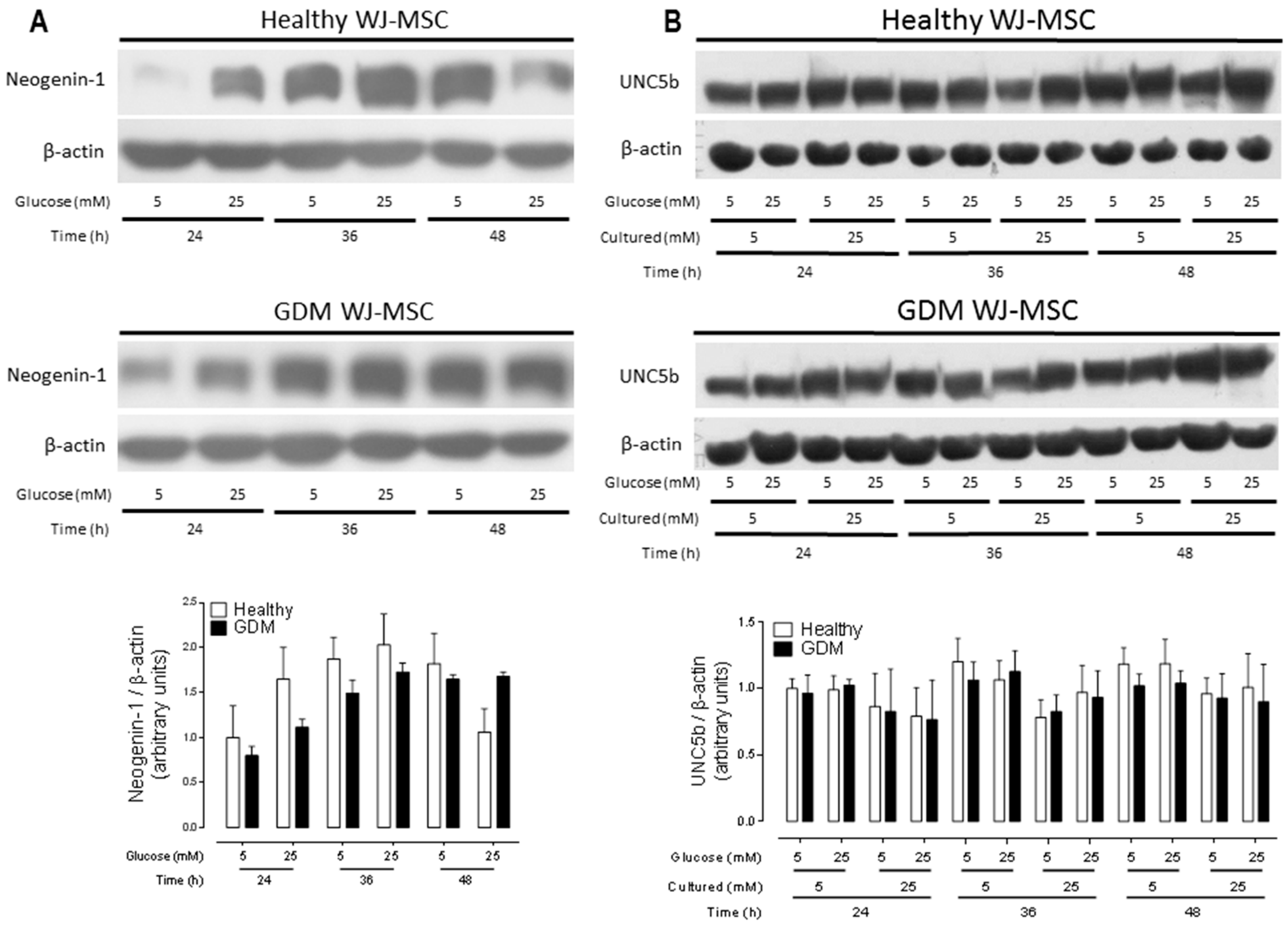

Figure S1. Protein levels of classical Netrin-1 receptors in normal and GDM WJ-MSC. ***A.*** Whole cell lysate was evaluated by Western blot for Neogenin-1 in WJ-MSC from normal and GDM cultures**.** β-actin was used as internal loading control. Exposure to different D-glucose concentration 5 - 25 mM for 24 - 48 hours. Data correspond to the mean ± S.E.M. (normal n=5, GDM n=3). ***B.*** Western blot for UNC5b in normal and GDM WJ-MSC**.** β-actin was used as internal loading control. Each quantified result corresponds to the mean ± S.E.M. (normal n=3, GDM n=6).

Table 2. Comparison of angiogenesis between healthy and GDM HUVEC.

**Healthy *vs. GDM***	***DMEM***	***EGM***	***CM 5 mM***	***CM 25 mM***	***CM 5 mM +2F5***	***CM 25 mM +2F5***
**DMEM**	*	n.s.	n.s.	n.s.	*	*
**EGM**	*	n.s.	n.s.	n.s.	*	*
**CM 5 mM**	*	n.s.	*	*	*	*
**CM 25 mM**	*	n.s.	*	*	*	*
**CM 5 mM + 2F5**	*	n.s.	n.s.	n.s.	*	*
**CM 25 mM + 2F5**	*	n.s.	n.s.	n.s.	*	*

Figure S2. Statistical analysis of different experimental conditions, comparing angiogenesis among healthy and GDM HUVEC. Left column correspond to normal HUVEC (N) and upper row correspond to GDM HUVEC. Data correspond to the mean ± S.E.M. (CM WJ-MSC and HUVEC, n=3). *$p < 0.05$ Normal vs. GDM, n.s. not significant.

A

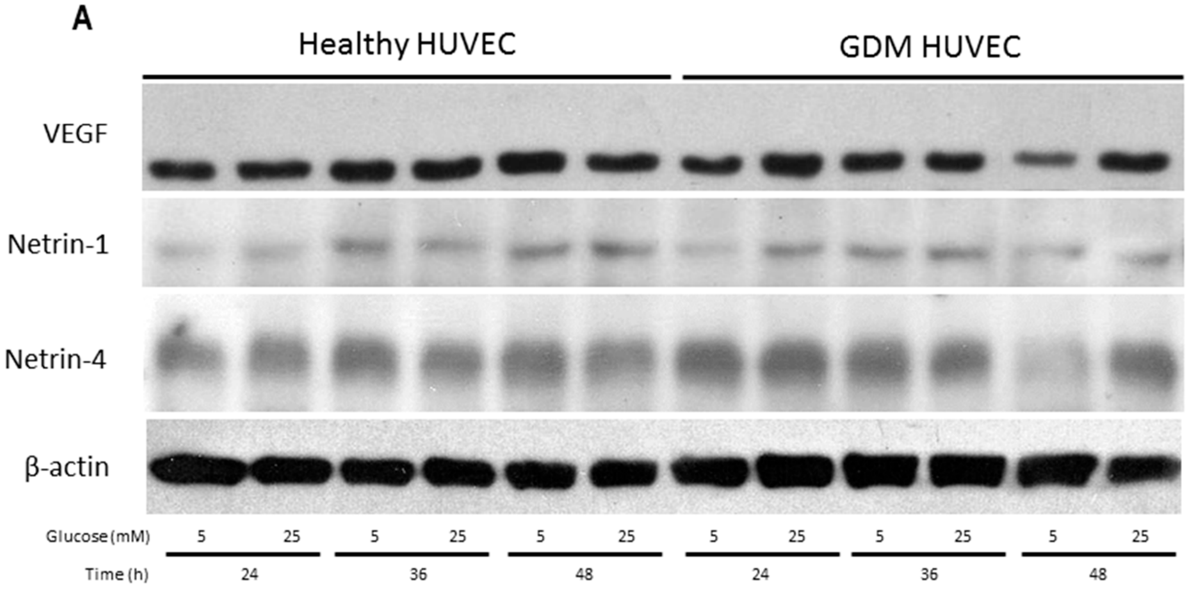

Healthy HUVEC
GDM HUVEC
VEGF
Netrin-1
Netrin-4
β-actin
Glucose (mM)
5
25
5
25
5
25
5
25
5
25
5
25
Time (h)
24
36
48
24
36
48

B

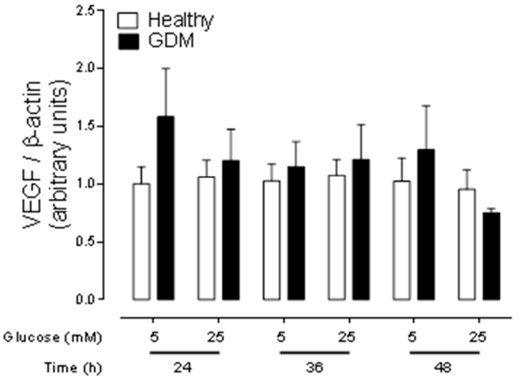

Healthy
GDM
VEGF / β-actin (arbitrary units)
0.0
0.5
1.0
1.5
2.0
2.5
Glucose (mM)
5
25
5
25
5
25
Time (h)
24
36
48

C

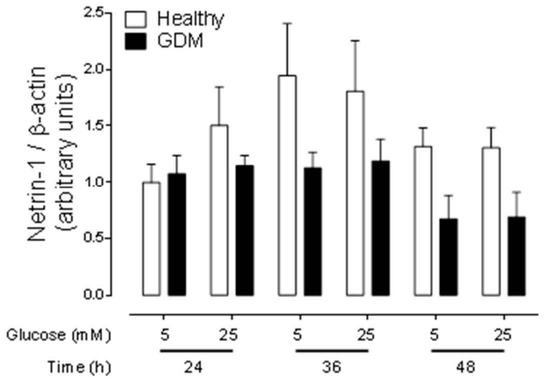

Healthy
GDM
Netrin-1 / β-actin (arbitrary units)
0.0
0.5
1.0
1.5
2.0
2.5
Glucose (mM)
5
25
5
25
5
25
Time (h)
24
36
48

D

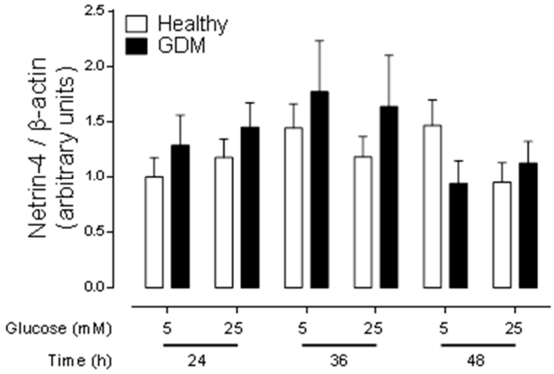

Healthy
GDM
Netrin-4 / β-actin (arbitrary units)
0.0
0.5
1.0
1.5
2.0
2.5
Glucose (mM)
5
25
5
25
5
25
Time (h)
24
36
48

Figure S3. Protein expression levels of canonical and non-canonical ligands in normal and GDM HUVEC. ***A.*** Whole cell lysate was evaluated by Western blot for VEGF, Netrin-1, Netrin-4 in HUVEC from normal and GDM cultures**.** β-actin was used as internal loading control. Exposure to different D-glucose concentration 5 - 25 mM for 24 - 48 hours. Data correspond to the mean ± S.E.M. (normal n=7, GDM n=3). ***B.*** Graph show quantified results for VEGF. ***C.*** Netrin-1 and ***D.*** Netrin-4.

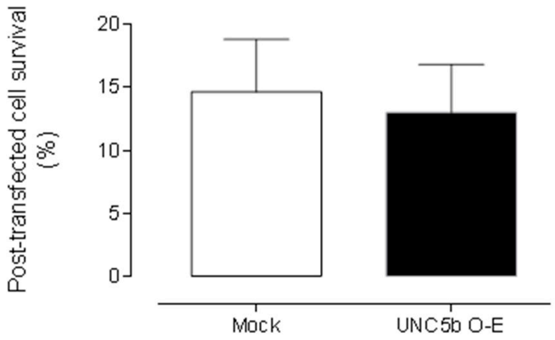

Figure S4. Cell survival after cell transfection. Healthy HUVEC was transfected with Mock over-expressing or UNC5b O-E construct (1µg/well) in primary cell medium 20% FBS, and cultured for 48 hours in normal HUVEC.
